# Conformational stability and activity analysis of two hydroxymethylbilane synthase mutants, K132N and V215E, with different phenotypic association with acute intermittent porphyria

**DOI:** 10.1042/BSR20130045

**Published:** 2013-08-08

**Authors:** Helene J. Bustad, Marta Vorland, Eva Rønneseth, Sverre Sandberg, Aurora Martinez, Karen Toska

**Affiliations:** *Department of Biomedicine, University of Bergen, 5009 Bergen, Norway; †Department of Global Public Health and Primary Care, University of Bergen, 5009 Bergen, Norway; ‡Norwegian Porphyria Centre (NAPOS), Laboratory of Clinical Biochemistry, Haukeland University Hospital, 5021 Bergen, Norway

**Keywords:** acute intermittent porphyria (AIP), genotype–phenotype relationships, hydroxymethylbilane synthase, ligand–protein interaction, porphobilinogen (PBG) deaminase, thermal stability, AIP, acute intermittent porphyria, ALA, δ-aminolaevulinic acid, DPM, dipyrromethane, DSF, differential scanning fluorimetry, DTT, dithiothreitol, eHMBS, erythroid hydroxymethylbilane synthase, HMB, hydroxymethylbilane, HMBS, hydroxymethylbilane synthase, HUS, Haukeland University Hospital, NAPOS, Norwegian Porphyria Centre, NPR, Norwegian Porphyria Registry, PBG, porphobilinogen, *T*_m_, half-denaturation temperature, u-ALA, urinary δ-aminolaevulinic acid, u-PBG, urinary porphobilinogen, wt, wild-type

## Abstract

The autosomal dominantly inherited disease AIP (acute intermittent porphyria) is caused by mutations in HMBS [hydroxymethylbilane synthase; also known as PBG (porphobilinogen) deaminase], the third enzyme in the haem biosynthesis pathway. Enzyme-intermediates with increasing number of PBG molecules are formed during the catalysis of HMBS. In this work, we studied the two uncharacterized mutants K132N and V215E comparative with wt (wild-type) HMBS and to the previously reported AIP-associated mutants R116W, R167W and R173W. These mainly present defects in conformational stability (R116W), enzyme kinetics (R167W) or both (R173W). A combination of native PAGE, CD, DSF (differential scanning fluorimetry) and ion-exchange chromatography was used to study conformational stability and activity of the recombinant enzymes. We also investigated the distribution of intermediates corresponding to specific elongation stages. It is well known that the thermostability of HMBS increases when the DPM (dipyrromethane) cofactor binds to the apoenzyme and the holoenzyme is formed. Interestingly, a decrease in thermal stability was measured concomitant to elongation of the pyrrole chain, indicating a loosening of the structure prior to product release. No conformational or kinetic defect was observed for the K132N mutant, whereas V215E presented lower conformational stability and probably a perturbed elongation process. This is in accordance with the high association of V215E with AIP. Our results contribute to interpret the molecular mechanisms for dysfunction of HMBS mutants and to establish genotype–phenotype relations for AIP.

## INTRODUCTION

The porphyrias are a group of rare metabolic disorders caused by malfunction of the enzymes in the haem biosynthesis pathway. Mutations in the third enzyme of this synthesis, HMBS [hydroxymethylbilane synthase; also known as PBG (porphobilinogen) deaminase, EC 2.5.1.61] are associated with the most frequently occurring acute porphyria, AIP (acute intermittent porphyria), an autosomal dominant inherited disease. Increased demand for haem, and thus an up-regulation of the haem biosynthesis, is induced by hormonal, environmental and metabolic factors among others. With deficient HMBS activity, an up-regulated haem biosynthesis results in accumulation of the haem precursors ALA (δ-aminolaevulinic acid) and PBG in tissues, which may trigger acute attacks (for reviews, see [1,2]). To date, more than 385 different mutations in the *HMBS* gene have been reported (available at http://www.hgmd.cf.ac.uk/ac/gene.php?gene=HMBS). Loss of important salt bridges or other intramolecular interactions because of mutations can lead to different effects on enzyme kinetics, stability, flexibility and dynamics of the enzyme. In this work, two uncharacterized Norwegian mutants, K132N and V215E, are compared with wt (wild-type) HMBS and the previously reported AIP-associated mutants R116W, R167W and R173W, which present defects mainly in conformational stability (R116W), enzyme kinetics (R167W) or both (R173W). See Supplementary Figure S1 (at http://www.bioscirep.org/bsr/033/bsr033e056add.htm) for a structural model and localization of the mutants.

Two HMBS isoforms have been reported in humans [3]; a housekeeping enzyme, referred to as HMBS (361 amino acids; M_w_ 39 330), which is ubiquitously expressed and active in all tissues, and an erythroid form [eHMBS (erythroid hydroxymethylbilane synthase), 344 amino acids; M_w_ 37 699] lacking 17 residues in the N-terminal. There seems to be no differences in *V*_max_ or *K*_m_ between the two isoforms [4]. The available crystal structure of human HMBS is valuable as a starting point to interpret the pathogenic mechanism for AIP-associated HMBS mutations [5,6] (PDB ID 3ECR), but it has not provided information on the 17 N-terminal residues that differ between the isoforms. The monomeric enzyme is organized in three domains (Supplementary Figure S1). Domain 1 (residues 1–116 and 216–239) and domain 2 (residues 117–215) are connected by a hinge region that introduce flexibility, whereas the hydrophobic cleft that is formed between the two domains defines the active site. Domain 3 (C-terminus, residues 240–361) holds a loop containing a conserved cysteine (Cys^261^) to which the DPM (dipyrromethane) cofactor binds covalently.

HMBS is thought to be expressed as an apoenzyme with a unique ability to assemble its own cofactor. The enzyme catalyses the assembly of four PBG molecules into the linear precursor of uroporphyrinogen III, HMB (hydroxymethylbilane, also called preuroporphyrinogen). Four enzyme–intermediate complexes emerge during this process (for a review, see [7]). The first three complexes (ES, ES_2_ and ES_3_) are relatively stable, whereas the fourth complex, ES_4_, is quickly hydrolysed into HMB [8]. The cofactor itself is not turned over and HMBS leaves the reaction as a holoenzyme [8,9]. The catalytic process of HMBS has been extensively studied [8,10–12], but the mechanism is still not fully understood. Several salt bridges and hydrogen bonds are established when the cofactor binds to HMBS, inducing additional interactions within the enzyme and cross-linking the domains [5,6,13]. These interactions lead to a stable structure that is resistant to both heat treatment and urea denaturation [14–16]. However, the subsequent condensation of the four PBG molecules is thought to induce conformational changes leading to an opening of the enzyme, giving space for the tetrapyrrole [6,12,13].

In this work, we aimed to further characterize malfunctioning mechanisms of HMBS mutants, and selected four natural mutations found in Norwegian AIP patients (R116W, R167W, R173W and V215E). In addition, K132N, detected in a patient presented with abdominal pain and indistinct porphyria-related biochemical findings, was included. We studied the conformational and thermal stability of recombinant wt HMBS and the five selected mutants. K132N, carrying a mutation located on the surface, far from the active site, has to our knowledge not previously been reported. V215E, although reported [17], has not been characterized. V215E is located in the hinge region between domains 1 and 2, close to the active site and is one of the most common AIP mutations in Norway. 59% of patients with this mutation experience porphyria attacks. The results of the novel mutants were validated by comparative analyses with the already characterized mutants R116W [15,18], R167W [19–21] and R173W [21–24]. These three mutations were chosen because of their different effects on the activity and conformational stability of HMBS. The kinetic failure of R116W can be associated with a strong conformational defect causing a severely misfolded enzyme unable to acquire the cofactor [5,25]. The misfolded R173W mutation has also been shown to be catalytically deleterious, and it is assumed that the substitution of the arginine residue will lead to an improper binding of cofactor and substrate, resulting in blocking of catalysis [23,26]. Furthermore, R167W is a catalytic mutant expected to have an inefficient elongation process, that consequently accumulates enzyme–intermediate complexes [6,27,28].

By using a combination of methods including native PAGE, enzyme kinetics, CD, DSF (differential scanning fluorimetry) and ion-exchange chromatography, we provide the comparative results on the wt HMBS and selected mutants that contribute to a better understanding of the elongation mechanism and the molecular basis of the dysfunctions of AIP associated mutations. R116W and R173W, which are the two mutants with the most severe phenotype, also present an almost total kinetic dysfunction that is largely related to their severe conformational instability. For the mutants R167W and V215E, which present a less severe phenotype, but yet a high association with AIP, the clinical profile appears to be related to a kinetic and a conformational instability, respectively. For K132N, no defect in enzyme kinetics, conformation or stability could be shown, which seems to fit with the lack of a clear phenotypic association with AIP for this mutant.

## EXPERIMENTAL

### Patients

Of all known porphyria patients in Norway, 70% are registered in the NPR (Norwegian Porphyria Registry) administered by the NAPOS (Norwegian Porphyria Centre). The register holds patients who have experienced symptoms or attacks because of their porphyria (designated *active*) as well as patients with *latent* porphyria who have never had symptoms that could be attributed to porphyria (mostly relatives of patients predictively examined by DNA sequence analysis). Each patient answers a disease-specific questionnaire, and information on diagnosis, symptoms, biochemical and genetic characteristics, is recorded. A total of 680 patients with AIP, all heterozygote, are registered in the NPR. Of these, 18 (15 active) have the R116W mutation, four patients (three active) are registered with R167W and another four (three active) with the R173W mutation. V215E is registered with 37 (22 active) patients in the NPR. The mutation K132N was discovered in a patient where sequencing of the *HMBS* gene was performed on the basis of abdominal pain and unclear porphyria-related biochemical findings. Following further biochemical studies, neither the index K132N patient nor the nine family members confirmed to have the Lys→Asn substitution have at any time presented biochemical findings consistent with a diagnosis of AIP.

Biochemical diagnosis of AIP was performed at the Laboratory of Clinical Biochemistry, HUS (Haukeland University Hospital). AIP has been established on the basis of increased u-ALA (urinary ALA) and u-PBG (urinary PBG) measured by ion-exchange chromatography [29] in relation to creatinine, and by reduced eHMBS activity measured in washed erythrocytes as described by Ford et al. [30]. DNA analyses were carried out at the Centre for Medical Genetics and Molecular Medicine, HUS.

The study was approved by the Regional Committee for Medical Research Ethics, and informed consent was gathered from all patients.

### Site-directed mutagenesis

The plasmid for protein expression of human HMBS was generously provided by Professor Pavel Martasek and Dana Ulbrichova as pGEX4T-1-expression vector [31]. The missense mutations R116W, K132N, R167W, R173W and V215E were introduced into HMBS cDNA using QuikChange® lightning mutagenesis kit (Stratagene). To verify the mutations, the entire gene was sequenced.

### Expression and purification of HMBS proteins

Wt and mutant HMBS were expressed in *Escherichia coli* BL21 (DE3) pLysS (Stratagene) as glutathione S-transferase fusion proteins. After inoculation with 50 ml overnight pre-cultures the cells were grown at 37°C in 950 ml Luria Bertani broth supplemented with 100 μM ampicillin, 34 μg/ml chloramphenicol and 2 g/l glucose. At *A*_600_~0.8 protein expression was induced by 1 mM IPTG (isopropyl thio-β-d-galactoside), grown overnight at 28°C, and harvested by centrifugation at 4°C, 4000 ***g*** for 15 min. Washed cells were subsequently resuspended in PBS (140 mM NaCl, 2.7 mM KCl, 10 mM Na_2_HPO_4_ and 1.8 mM KH_2_PO_4_), pH 7.4, containing 1 mM EDTA, 0.5 mM PMSF, 1 mM benzamidine and protease inhibitor (cOmplete ULTRA tablet, Roche Applied Science), and sonicated on ice. The sonicate was centrifuged at 14 000 ***g*** for 45 min at 4°C, before the supernatant was loaded onto a glutathione-sepharose 4B column (GE Healthcare), and washed with 500 ml PBS containing 1 mM EDTA, followed by two column volumes of only PBS. The thrombin (200 units/l) digest was performed by gently shaking at 4°C for 2 h in 50 mM Tris–HCl, pH 8.0, containing 2 mM CaCl_2_, 1 mM DTT (dithiothreitol) and 150 mM NaCl. The final purified proteins, in 50 mM Tris–HCl, pH 8.0, were up-concentrated (>10 mg/ml) with 30 kDa cut-off filters (Amicon Ultra centrifugal filters, Millipore), aliquoted and stored in liquid N_2_ until use. The purity of the protein was confirmed by SDS–PAGE.

### Enzymatic activity assay of recombinant HMBS

The enzymatic activity of recombinant HMBS was assayed in 50 mM Na-Hepes, pH 8.2, containing 0.1 M DTT. Approx. 5 μg enzyme was pre-incubated for 3 min at 37°C and the enzyme reaction was initiated by the addition of 100 μM preheated PBG solution. After 4 min the reaction was terminated by the addition of 5 M HCl and benzoquinone (0.1% in methanol) and samples were incubated on ice for 30 min, protected from light, before the absorbance was determined at 405 nm. The activity of recombinant HMBS was defined as nmol of uroporphyrinogen I/h per mg of enzyme at 37°C under the given assay conditions. *K*_m_ and *V*_max_ were determined using varying PBG concentrations ranging from 1.5 μM to 4 mM. Protein concentrations were determined from the theoretical molar extinction coefficients (ϵ_280_=0.39 for wt, V215E and K132N, and ϵ_280_=0.54 for R116W, R167W and R173W, for a 0.1% solution) [32]. The kinetic parameters were obtained by non-linear curve fitting using Sigma Plot 11.0 with the additional module EKWizard 1.2.0.0.

### Anion-exchange chromatography

The enzyme (~1 mg of wt or ~3 mg of V215E) was applied to a MiniQ 4.6/50 PE column (GE Healthcare) equilibrated in 50 mM Tris–HCl, pH 8.2 at 4°C, attached to a Bio-Rad Biologic Duo Flow FPLC system. Elution was performed using a pre-programmed NaCl gradient (0–400 mM NaCl) with collection of 200 μl fractions, and the absorbance was monitored at 280 nm. The individual peaks were collected and up-concentrated using Amicon Ultra 30 kDa cut-off centrifugal filters (Millipore) for further analysis by DSF.

### Polyacrylamide gel electrophoresis

10% (w/v) SDS- and native PAGE were both performed in a discontinuous system (stacking gel buffer pH 6.8 and running gel buffer pH 8.8) with Tris–glycine tank buffer (pH 8.3) at room temperature (22°C) [33]. Native PAGE was performed without denaturing agents (SDS, 2-mercaptoethanol) and samples were not heated prior to loading [16]. Approx. 5 and 10 μg enzyme were applied for SDS–PAGE and native PAGE, respectively.

### Circular dichroism

A Jasco J-810 spectropolarimeter equipped with a PTC-348WI Peltier element for temperature control at 25°C and a 300 μl quartz cell with a path length of 1 mm were used for far-UV and thermal denaturation CD measurements. The HMBS samples (final concentrations 5–9 μM) were prepared in 10 mM K-phosphate, pH 8.2, 150 mM KF. Four scans were accumulated for each spectrum and buffer scans were subtracted, using the accompanying software from Jasco. Thermal denaturation profiles were obtained by recording the ellipticity at 222 nm as a function of temperature between 20 and 95°C (2°C/min). The far-UV and thermal scans were smoothed using a negative exponential algorithm with a sample proportion=0.05 and polynomial degree=2 (1 for thermal scans). CDNN [34] was used to estimate the secondary structure content. The thermal scans were normalized and curve fitted to a two-state unfolding model [35] and converted to fraction of unfolded protein [36]. The slope was fixed to zero (md=0) for post-transitional baseline.

### Differential scanning fluorimetry

A LightCycler 480 Real-Time PCR System (Roche Applied Science) was used for the fluorimetric thermal denaturation scans. 50 μl of either 2.5 μM HMBS or mutants in 10 mM K-phosphate, pH 8.2, 150 mM KF and 5× SYPRO Orange (Molecular Probes, Inc.) were added to each well of a 96-well plate (Roche Applied Science). The plates were incubated for 30 min at room temperature prior to thermal scans. The increase in SYPRO Orange fluorescence is associated with protein unfolding (λ_ex_=465 nm, λ_em_=610 nm) and was used for monitoring the thermal denaturation. The thermal scans were performed at a scan rate of 2°C/min from 20 to 95°C with data points collected approximately every 0.2°C. The data were scaled to reflect the fraction of unfolded protein. *T*_m_ (half-denaturing temperature)-values were determined at fraction of unfolded=0.5, as well as from the maxima of the first derivative.

Titration experiments by DSF were performed under identical conditions as above, except that 1, 25, 50 or 100 μM PBG was added to wt or mutants. R167W was additionally titrated with 500 and 1000 μM PBG.

## RESULTS AND DISCUSSION

### Patient data

The mean values of eHMBS activity, u-ALA and u-PBG were within the reference limits for the patients with the K132N mutation. The mean levels of eHMBS activity for the other four mutations were all below the lower reference limit. The results are summarized in Table 1. The analyses indicate a very severe phenotype for the patients with the R116W and R173W mutations, and severe for R167W and V215E. The biochemical analyses did not reveal any effect of the K132N mutation on the biochemical phenotype.

**Table 1:** eHMBS activity, and u-ALA and u-PBG concentrations in AIP patients with the selected HMBS mutations, compared with healthy individuals

		eHMBS (units/l)*		u-ALA/creatinine (μmol/mmol)		u-PBG/creatinine (μmol/mmol)	
Mutation	No. of patients (number active)	Range	Mean	Range	Mean	Range	Mean
Healthy†	−	0.43–0.89	−	<5.0	−	<0.8	−
R116W	18 (15)	0.20–0.41	0.29	1.52–78.39	10.48	0.33–46.05	12.31
K132N	10 (0)	0.32–0.61	0.46	2.08–5.99	2.93	0.32–0.54	0.43
R167W	4 (3)	0.28–0.42	0.36	2.41–8.21	5.62	0.35–8.47	5.39
R173W	4 (3)	0.19–0.27	0.24	1.85–3.27	2.60	0.40–1.21	0.92
V215E	37 (22)	0.16–0.61	0.32	1.20–19.02	5.18	0.31–33.89	5.67

*One unit of HMBS activity is 1 μmol of uroporphyrinogen I/min.

^†^Reference values compiled at HUS.

### Expression of recombinant HMBS and mutants

**Figure 1:**
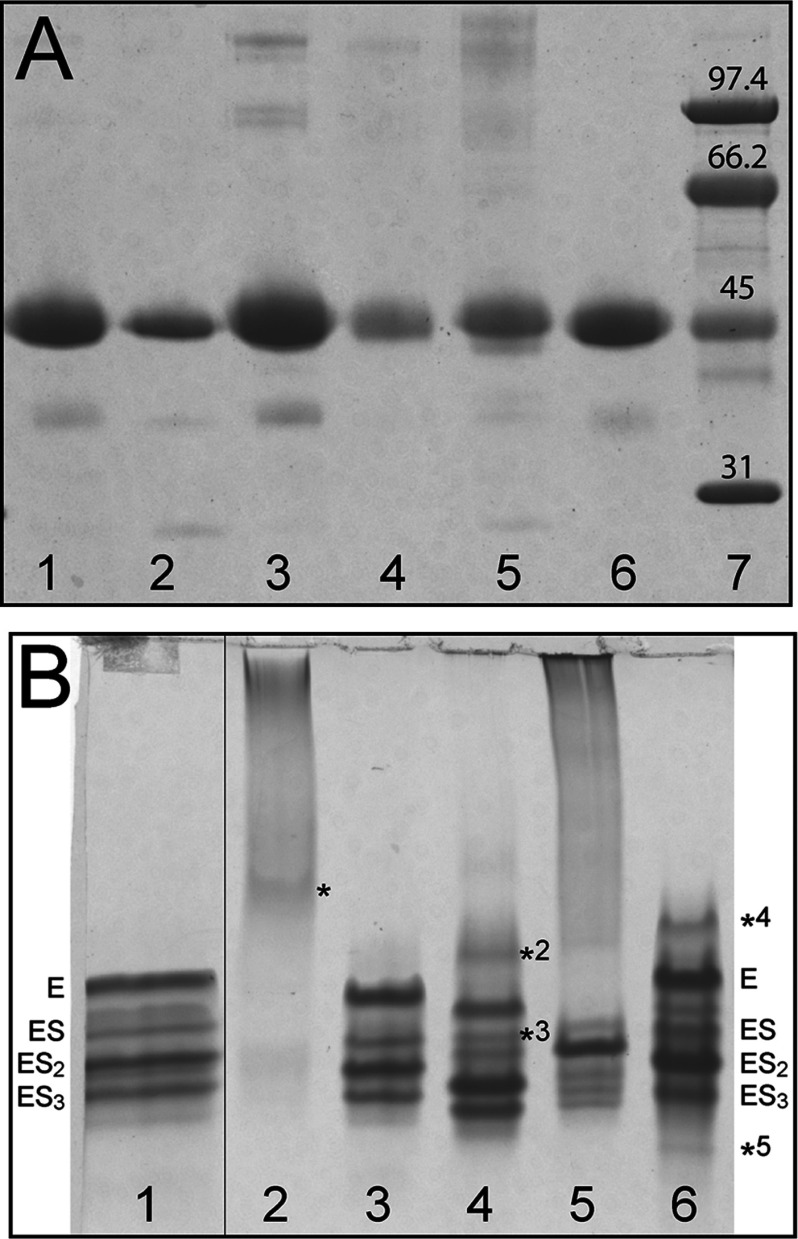
SDS–PAGE and native PAGE of wt and mutant HMBS (**A**) SDS–PAGE showing recombinant HMBS as purified: Wt (lane 1), R116W (lane 2), K132N (lane 3), R167W (lane 4), R173W (lane 5) and V215E (lane 6). Low molecular mass SDS–PAGE standards (lane 7). (**B**) Native PAGE of recombinant HMBS as purified: Wt (lane 1), R116W (lane 2), K132N (lane 3), R167W (lane 4), R173W (lane 5) and V215E (lane 6). See the main text for the interpretation of protein bands.

To study the effect of the newly discovered missense mutations K132N and V215E, together with R116W, R167W and R173W, wt and mutant HMBS were expressed in *E. coli* and purified with high yield (≥50 mg wt and mutant HMBS were obtained from 1 l culture) and purity (>95% pure) as seen by SDS–PAGE (Figure 1A). All purified proteins showed distinct SDS–PAGE bands, corresponding to approximately 44 kDa.

To analyse the enzyme–intermediate complexes the purified enzymes were separated by native PAGE [14]. Previous reports on native PAGE analyses have shown an inconsistent number of bands for wt HMBS [6,28,37]. In this work, we found four major bands (Figure 1B), as also reported recently [37]. Variations in recombinant enzyme purification protocols may lead to loss of enzyme–intermediate complexes [8,38]. The successive increase in negative charge of the intermediates is a result of increased number of PBGs, and the highest band in wt HMBS (Figure 1B, lane 1) is thus interpreted to be the holoenzyme (E) [37]. Following downwards, the bands have been associated with ES (containing one PBG monomer substrate in addition to the DPM cofactor), ES_2_ (two PBGs) and ES_3_ (three PBGs) [37]. All the mutations included in this work are expected to change the charge of the protein at the given pH (8.3) and will give a slightly different maximal migration for the intermediates. Except for K132N, which revealed a similar pattern of bands as wt, all the mutants showed differences in the distribution of bands (Figure 1B). R116W failed to provide discrete bands, although a weak band may be distinguished (* in lane 2; Figure 1B). Based on the instability of this mutant (see below), and its secondary structure content which resembles that of the wt apoenzyme [39] (see also below), this band may represent a cofactor-free apoform of mutant HMBS [38]. R167W shows a more complex pattern than wt HMBS with an additional faint band (*^2^ in lane 4; Figure 1B) migrating above the E band, possibly representing a partially formed holoenzyme (Figure 1B). Moreover, this mutant also presents a discrete band between E and ES (*^3^), which is also observed as a minor band for wt (lane 1; Figure 1B), and might correspond to a non-identified catalytic intermediate. The unstable R173W (lane 5, Figure 1B) migrated mainly as one band, whereas V215E (lane 6, Figure 1B) showed two additional bands to those observed in wt. The band denoted with *^4^ would correspond to *^2^ for R167W, and *^5^ might indicate an accumulation of the ES_4_ intermediate prior to product release. The ES_4_ intermediate is normally not observed [8] other than in situations with slow or no release of the product [40]. The accumulation of enzyme–intermediates seems to indicate some disturbance in the elongation mechanism which is probably related to the misfolding and instability of V215E (see below).

### Kinetics parameters of wt HMBS and mutants

**Figure 2:**
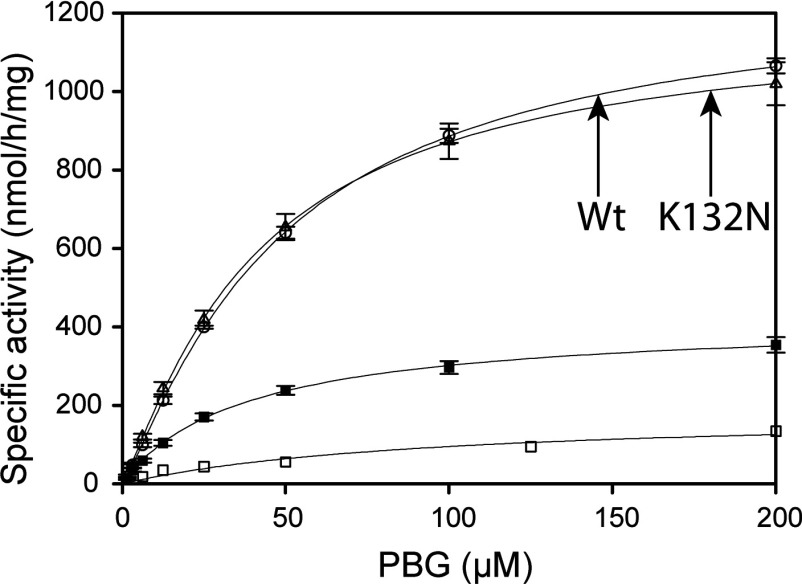
The catalytic activity of wt HMBS and mutants as a function of the substrate (PBG) concentration Activity is measured as uroporphyrinogen I formation at 37°C. Wt (○, *n*=8), K132N (∆, *n*=8), R167W (□, *n*=12), and V215E (■, *n*=10).

The steady-state kinetic characterization of the recombinant wt and mutant enzymes presented here revealed significantly reduced (<5%) relative activity at standard conditions for R116W, R167W and R173W (Table 2), as expected [15,21,41]. The low activity of R116W and R173W precluded the determination of their kinetic parameters. On the other hand, for R167W the large kinetic defect seems to be associated with a very high *K*_m_ and decreased *V*_max_ (Figure 2, Table 2) as expected from an inefficient elongation process, as described for the R167Q mutant [28]. No loss in relative activity at standard conditions or change in *V*_max_ and *K*_m_, compared with wt HMBS, was measured for K132N (Figure 2), indicating no apparent kinetic dysfunction. The V215E mutation caused a 70% loss in both activity and *V*_max_; however, *K*_m_ was unchanged (Figure 2, Table 2).

**Table 2:** Steady-state kinetic parameters of recombinant wt HMBS and mutants

Mutation	Relative activity* (%)	*V*_max_ (nmol/h per mg)†‡	*K*_m_ (PBG) (μM)†
Wt	100	1261±75	48±5
R116W	0.5	n.a.	n.a.
K132N	97	1182±78	41±6
R167W	4.2	615±21	1579±111
R173W	0.6	n.a.	n.a.
V215E	30	423±36	39±9

*Relative activity was measured at standard conditions: wt and mutant HMBS (~5 μg) and 100 μM PBG for a reaction time of 4 min at 37°C. The same results were obtained after 20 min incubation.

^†^*V*_max_ and *K*_m_ were calculated using fitting of the data to Michaelis–Menten kinetics.

^‡^The activity of HMBS was defined as nmol of uroporphyrinogen I/h per mg of enzyme at 37°C under the given assay conditions.

### Conformational and thermal stability studies

**Figure 3:**
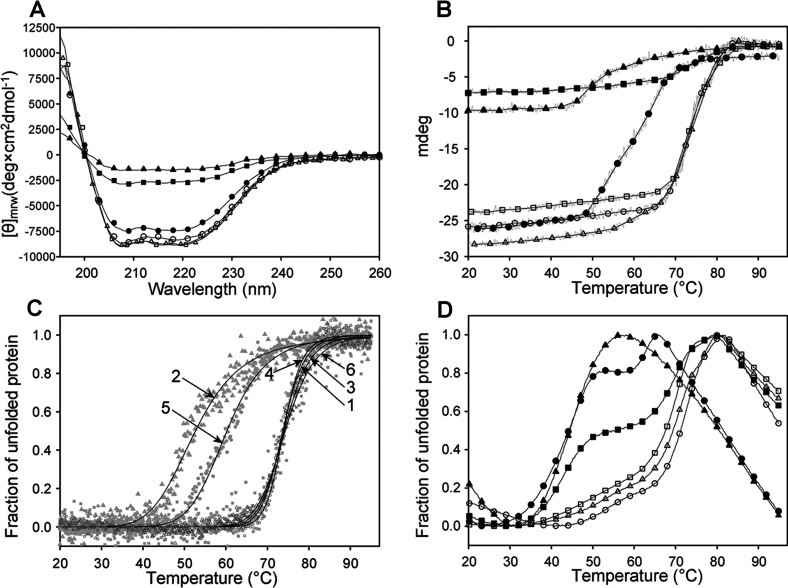
Conformational stability of wt HMBS and mutants, as studied by CD and DSF (**A**) Far-UV CD of wt (○), R116W (▲), K132N (∆), R167W (□), R173W (●) and V215E (■). [θ], mean residue ellipticity. (**B**) The CD-monitored thermal denaturation, presented as mdeg at 222 nm *versus* temperature. (**C**) The CD-monitored thermal denaturation, normalized to fraction of unfolded protein, and curve fitting to a two-state transition model for wt (1), R116W (2), K132N (3), R167W (4), R173W (5) and V215E (6). (**D**) Thermal denaturation monitored by DSF for wt (○), R116W (▲), K132N (∆), R167W (□), R173W (●) and V215E (■). A representative plot of each of four parallels is shown in the figure. The *T*_m_-values obtained from (**C**) and (**D**) are summarized in Table 4.

To better understand the decreased activity of most of the mutants, CD and DSF were applied to investigate the conformation and thermal stability compared with the wt. The far-UV spectrum of wt (Figure 3A) showed two local minima at 208 and 222 nm, and provided a content in α-helical and β-sheet structure in accordance with the X-ray crystal structure of the human enzyme [42,43]. The secondary structure content estimated from the far-UV spectra is given in Table 3. Similar results were obtained for K132N and R167W. A mild conformational defect was revealed, however, for R173W, while the large loss of ellipticity at equal protein concentrations observed for R116W and V215E was concurrent with largely decreased α-helical content, a feature characteristic of the apoenzyme of *E. coli* HMBS [39]. Native PAGE supported an unstable apoenzyme conformation for R116W [44], whereas for V215E a putative species corresponding to the partially formed holoenzyme (*^4^) may also be accompanying the stable intermediates (Figure 1B). The unfolding profiles from temperature-dependent CD shown in Figure 3(B) exhibit the loss of ellipticity for R116W and V215E, as also seen by far-UV. Normalized unfolding profiles of wt and mutants were fitted to a two-state transition model (Figure 3C) and *T*_m_-values were extracted, further revealing the large conformational instability of R116W and R173W. For wt HMBS the *T*_m_ obtained was very high, i.e. *T*_m_~74°C (Table 4), as expected from the high thermal stability of the enzyme activity [14,15,45]. R116W and R173W displayed lower *T*_m_-values, approx. 53 and 60°C, respectively (Figure 3C, Table 4), reflecting their lower thermal stability, whereas K132N and R167W showed stability similar to that of wt. For the V215E mutant, despite the decrease in ellipticity observed by CD (Figures 3A and 3B), temperature-dependent CD provided *T*_m_ similar to wt. Further insight on this mutant, comparative to the wt and the other mutant forms was obtained by DSF.

**Table 3:** The content of secondary structure in wt HMBS and mutants estimated from far UV CD spectra Secondary structure is estimated from far-UV CD spectra using the CDNN algorithm [34].

Mutation	α-helix (%)	β-sheet (%)
Wt	26.7±0.3	23.4±0.3
Wt (X-ray)*	31.2	19.8
R116W	16.2±0.1	41.9±0.4
K132N	27.0±0.7	24.5±1.5
R167W	28.4±0.3	21.8±0.3
R173W	24.9±0.3	26.6±0.5
V215E	17.7±0.2	38.3±0.6

*Determined from the crystal structure of the human enzyme (PBD 3ECR) using the DSSP algorithm [42,43].

**Table 4:** The *T*_m_ for wt HMBS and mutants as measured by CD and DSF

	*T*_m_-values (°C)			
Mutation	CD*	DSF†	DSF, *T*_m1_‡	DSF, *T*_m2_‡
Wt	74.0±0.1	71.1±0.6	54.4±0.8§	72.8±0.3
R116W	53.1±0.4	42.6±2.1	42.1±1.7	−
K132N	74.1±0.3	69.4±0.3	53.3±0.5§	71.0±0.2
R167W	74.3±0.0	67.8±0.2	48.2±0.9§	70.6±0.1
R173W	60.3±0.2	44.5±0.6	45.6±0.4	62.8±0.1
V215E	74.5±0.3	54.7±2.8	43.2±0.5	70.3±0.4

*Determined at fraction of unfolded=0.5 by curve fitting of the thermal transitions [35,36]. See Figure 3(B).

^†^Determined at fraction of unfolded=0.5 in the thermal transitions. See Figure 3(C).

^‡^*T*_m1_ and *T*_m2_ were calculated from the first derivative using the transitions observed in the temperature scans. See Figure 3(D).

^§^Minor transitions.

DSF (Figure 3D) largely confirmed the results from the CD measurements for wt, R116W, K132N, R167W and R173W, although the *T*_m_-values were lower than those obtained by temperature-dependent CD, as summarized in Table 4. Moreover, DSF provided evidence for two unfolding transitions for wt and all mutants, except for the most unstable R116W, and hence gave a more detailed insight into the conformational stability of the enzyme–intermediate components in each preparation than temperature-dependent CD. In particular, DSF revealed a large enzyme population with reduced stability (*T*_m_=43°C) in V215E (Figure 3D, Table 4). Both temperature-dependent CD and DSF monitor the unfolding transitions for the overall 3D structure, where thermal CD at 222 nm follows the unfolding of the secondary structure, notably α-helical, and DSF displays the solvent exposure of hydrophobic patches. [46]. Although the profiles from CD and fluorescence methods are coincident for numerous proteins, different thermal denaturation profiles, and consequently *T*_m_-values, might be obtained from these two methods, as has often been observed [47–49]. In order to interpret the obtained thermostability data for wt and mutants as isolated we performed stability studies with PBG saturation (see below).

While the kinetic failure in R116W is associated with a misfolded enzyme unable to acquire cofactor, this is not the case for R173W, which also includes a population of properly folded enzyme as supported by native PAGE, CD and DSF. Moreover, this mutation appears to directly affect the binding of substrate and the elongation mechanism, resulting in accumulation of mainly one intermediate (lane 5; Figure 1B). R167W, on the other hand, is a representative kinetic mutation [6,28], with its conformational change having little effect on the stability of the protein.

### HMBS is destabilized upon substrate saturation

**Figure 4:**
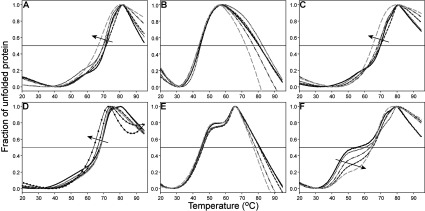
Thermal stability of wt HMBS and mutants with increasing concentration of PBG 0 μM (solid line), 1 μM (**·····**), 25 μM (---), 50 μM (**··**-**··**) or 100 μM (– –) PBG was added to 2 μM wt HMBS or mutants. For R167W, addition of 500 μM (**●)** and 1000 μM (▲) PBG were also analysed. (**A**) For wt HMBS, a Δ*T*_m_−4.5°C was measured at saturating concentration of PBG. (**B**) R116W, Δ*T*_m_~0°C. (**C**) K132N, Δ*T*_m_~−4.5°. (**D**) R167W, Δ*T*_m_~0°C (1–100 μM PBG). 500 and 1000 μM PBG gave Δ*T*_m_~−1.5°C and −4°C, respectively. (**E**) R173W Δ*T*_m_~0°C. (**F**) V215E was the mutant most affected upon titration with PBG. The first transition (arrow) was clearly stabilized (Δ*T*_m_~14°C) upon saturation with PBG, whereas the second transition was only slightly destabilized (Δ*T*_m_~−2°C).

The thermostability of wt and mutant HMBS was further analysed by DSF in the presence of increasing amounts of the substrate PBG (Figure 4). An increase in thermodynamic stability is usually seen upon ligand binding [50], and the binding of the DPM cofactor to the apoenzyme triggers a conformational change to a more compact enzyme [5,6,39]. However, when saturated with 20-fold substrate (100 μM PBG) both wt HMBS and K132N produced a shift in *T*_m_-values (∆*T*_m_=4.5°C) towards lower temperature (Figures 4A and 4C). The decreased stability may reflect an overall change in the equilibrium within the intermediates, towards less stable species. A tendency towards a more homogeneous enzyme sample was also seen, as the two observed transitions collapse into one at the highest substrate concentration. Heat treatment in this assay at high concentration of substrate may, however, induce a redistribution of the enzyme–intermediate complexes because of on-going catalysis [8]. Nevertheless, loss in stability with an increasing number of substrates appears to be in agreement with the suggested opening and loosening of the enzyme structure in order to accommodate the hexapyrrole and to prepare for product release [6,12,13]. Neither R116W nor R173W were affected by the addition of, and saturation with, PBG (Figures 4B and 4E), a fact supported by the kinetic analyses. These results agree with previous reports on the lack of proper cofactor or substrate binding and depleted activity of mutants with substitutions at Arg^116^ [15,26] and Arg^173^ [4,21]. R167W only revealed a decrease in *T*_m_ when incubated with high PBG concentrations (500 and 1000 μM) (Figure 4D), in agreement with the high *K*_m_-value, indicative of a low affinity for the substrate (Table 2). V215E, however, showed a rather different denaturation profile with a noticeable change in distribution with increasing PBG concentration (Figure 4F) where the transition with the lowest stability was shifted towards higher *T*_m_. The most stable population (close to wt in *T*_m_, see Table 4) was less affected. Higher PBG-concentrations (up to 4 mM, results not shown) did not further affect the denaturation profiles or V215E activity in agreement with a wt-like *K*_m_ (Table 2) indicating that the folding defect is the main contributor to the impaired mechanism of this mutant.

**Figure 5:**
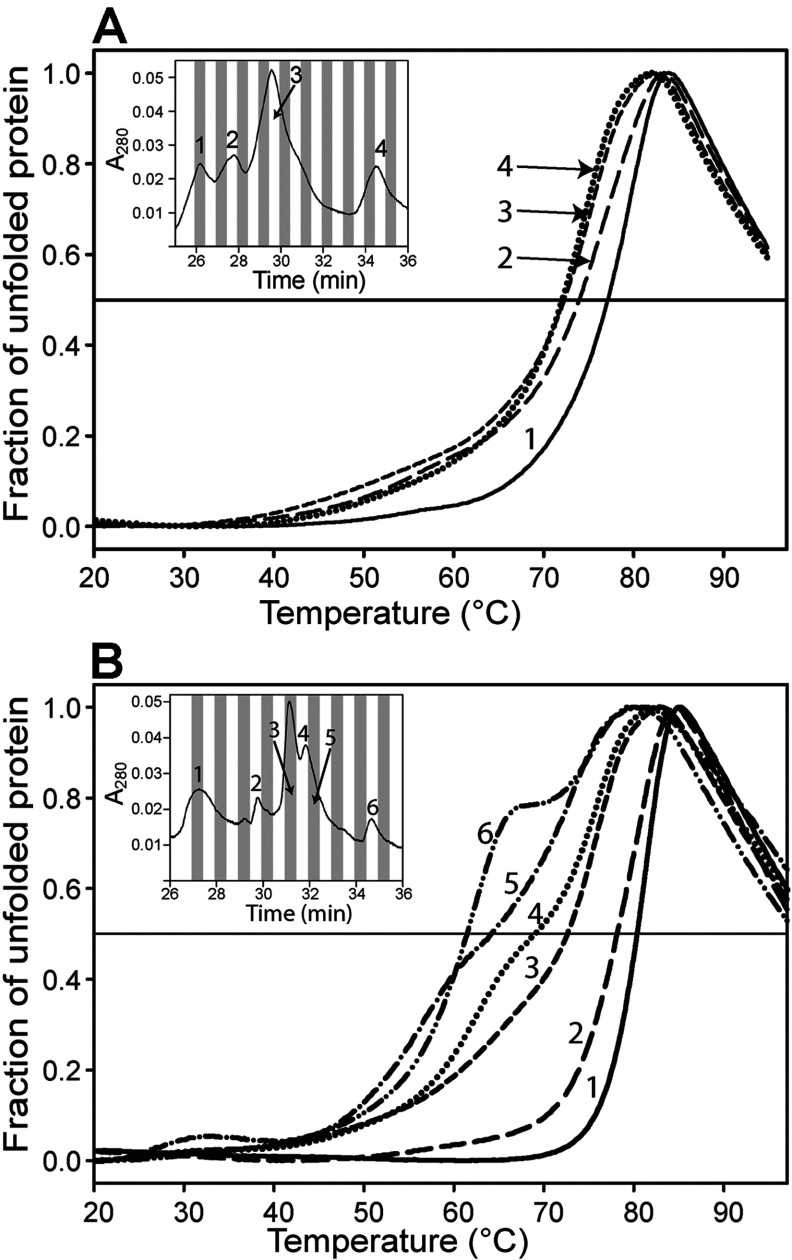
DSF of the fractionated peaks obtained from ion-exchange chromatography of wt HMBS and the V215E mutant (**A**) For wt HMBS, the unfolding transitions show decreasing thermal stability with increasing retention time, indicating that the stability is decreased with increasing amount of bound PBG-molecules (E, peak 1; ES, peak 2; ES_2_, peak 3; ES_3_, peak 4). ∆*T*_m_ is~−5°C from peak 1 to peak 4. The inset shows the ion-exchange chromatography profile. (**B**) For V215E, the ion-exchange chromatography profile shows a rather similar pattern to that of wt, but further investigation with DSF reveals a wider span of thermal stability (∆*T*_m_=−18.5°C) for the fractions obtained for this mutant.

The four bands observed in native PAGE for the purified wt HMBS (lane 1; Figure 1B) were fractionated by anion-exchange chromatography. In addition, we investigated the thermal denaturation of each fractionated peak by DSF. Initially, each peak was analysed by native PAGE (results not shown) and correlated to the major species in Figure 1(B), being E (holoenzyme, peak 1), ES (peak 2), ES_2_ (peak 3) and ES_3_ (peak 4) (Figure 5A), as previously reported [37]. The thermal stability of the intermediate complexes revealed that increasing amounts of bound substrate decreased the overall thermal stability of wt HMBS (Figure 5A). Analysis of K132N provided similar results to wt (results not shown) whereas V215E displayed a much larger shift between the peaks with the shortest and the longest retention times (∆*T*_m_=−18.5°C) (Figure 5B). This is in agreement with the complex DSF profile for the unfractionated preparation (Figures 3D and 4F). Nevertheless, most of the less stable population seen by DSF (Figure 3D), which probably correspond to the fraction *^4^ (lane 6, Figure 1B), is not observed in the fractions, and was most likely lost through aggregation and interaction with the chromatographic material during anion-exchange chromatography.

### Conclusion

In this work, we have acquired knowledge on the conformational stability of wt HMBS and selected mutants, as well as gained insight into the elongation process of the HMBS catalysis. The results also contribute to the understanding of the phenotype-genotype correlations in AIP. R116W and R173W, the two mutants with the most severe phenotype, present an almost total kinetic dysfunction which is largely associated with their severe conformational instability. R167W presents a less severe phenotype yet high association with AIP, and the clinical profile is mainly associated with a catalytic disruption. The novel mutation investigated in this work, K132N, has an unclear clinical significance, and our results show similar kinetic, conformational and thermal stability to wt HMBS, supporting that this mutation does not induce or cause classical AIP symptoms or biochemical findings. Lys^132^ is localized on the surface of domain 2, away from the active site, and is most likely not directly involved in the synthesis of HMB. The previously reported, although uncharacterized, V215E is located in the vicinity of the active site, and is part of a hinge region connecting domains 1 and 2. Although not interacting directly with the cofactor, the substitution of a hydrophobic Val with a negatively charged Glu residue in this position appears to alter intramolecular interactions, affecting the folding and stability of the enzyme, and the faulty mechanism of V215E appears to be the result of the folding defects. There seems to be a close relationship between the biochemical characteristics of the isolated recombinant enzyme, the biochemical phenotype, and the AIP association. The patients are highly affected, with 59% experiencing attacks, and our findings correlate with the high disease association.

Our results contribute to increasing our knowledge and understanding of the complete biochemical mechanism and the molecular basis for the phenotypic outcome of HMBS mutants, which is important in the future investigation of treatments for AIP.

## Online data

Supplementary data
