# Supplementary material for: Conformational stability and activity analysis of two hydroxymethylbilane synthase mutants, K132N and V215E, with different phenotypic association with acute intermittent porphyria
Source: Biosci Rep. 2013 Aug 8;33(4):e00056. doi: 10.1042/BSR20130045 (PMC3738108; doi:10.1042/BSR20130045)
Supplement: Supplementary data [file bsr033e056add.pdf]

Biosci. Rep. (2013) / **33** / art:e00056 / doi 10.1042/BSR20130045

SUPPLEMENTARY DATA

Conformational stability and activity analysis of two hydroxymethylbilane synthase mutants, K132N and V215E, with different phenotypic association with acute intermittent porphyria

Helene J. BUSTAD*†[1], Marta VORLAND‡, Eva RØNNESETH‡, Sverre SANDBERG†, Aurora MARTINEZ* and Karen TOSKA‡

*Department of Biomedicine, University of Bergen, 5009 Bergen, Norway, †Department of Global Public Health and Primary Care, University of Bergen, 5009 Bergen, Norway, and ‡Norwegian Porphyria Centre (NAPOS), Laboratory of Clinical Biochemistry, Haukeland University Hospital, 5021 Bergen, Norway

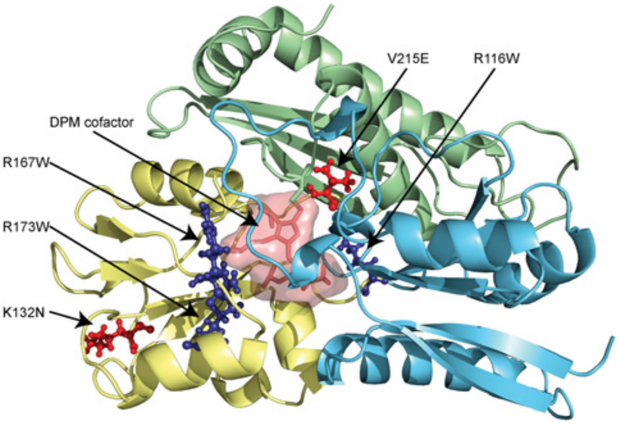

Figure S1 Structural model of HMBS
The location of the two novel mutations studied in this work, K132N and V215E, are presented as ball-and-sticks in red, whereas the three previously reported mutations (R116W, R167W and R173W) are presented as ball-and-sticks in dark blue. The complete structural model of HMBS was constructed with Modeller [1] based on multiple templates (PBD IDs: 1AH5, 1PDA, 1GTK, 1YPN, 3ECR and 3EQ1). Coordinates for residues 55–75 have been issued to loop optimization constructing a large set of possible confirmations. The lowest energy structure was chosen, and further refined with energy minimization using Amber 10 [2].

REFERENCES

1 Fiser, A. and Sali, A. (2003) Modeller: generation and refinement of homology-based protein structure models. Methods Enzymol. **374**, 461–491

2 Case, D. A., Cheatham, 3rd, T. E., Darden, T., Gohlke, H., Luo, R., Merz, Jr, K. M., Onufriev, A., Simmerling, C., Wang, B. and Woods, R. J. (2005) The Amber biomolecular simulation programs. J. Comput. Chem. **26**, 1668–1688

Received 19 April 2013/23 May 2013; accepted 29 May 2013

Published as Immediate Publication 2 July 2013, doi 10.1042/BSR20130045

[1] To whom correspondence should be addressed (email helenejbustad@gmail.com).

© 2013 The Author(s) This is an Open Access article distributed under the terms of the Creative Commons Attribution Licence (CC-BY) (http://creativecommons.org/licenses/by/3.0/) which permits unrestricted use, distribution and reproduction in any medium, provided the original work is properly cited.
